# Investigation of Position Sensing and Energy Harvesting of a Flexible Triboelectric Touch Pad

**DOI:** 10.3390/nano8080613

**Published:** 2018-08-13

**Authors:** Tao Chen, Qiongfeng Shi, Kunpu Li, Zhan Yang, Huicong Liu, Lining Sun, Jan A. Dziuban, Chengkuo Lee

**Affiliations:** 1 Jiangsu Provincial Key Laboratory of Advanced Robotics, School of Mechanical and Electric Engineering & Collaborative Innovation Center of Suzhou Nano Science and Technology, Soochow University, Suzhou 215123, China; chent@suda.edu.cn (T.C.); yangzhan@suda.edu.cn (Z.Y.); lnsun@hit.edu.cn (L.S.); 2 Department of Electrical and Computer Engineering, National University of Singapore, 4 Engineering Drive 3, Singapore 117576, Singapore; qiongfeng@u.nus.edu (Q.S.); li.kunpu@u.nus.edu (K.L.); 3 Center for Intelligent Sensors and MEMS, National University of Singapore, E6 #05-11F, 5 Engineering Drive 1, Singapore 117608, Singapore; 4 Hybrid-Integrated Flexible (Stretchable) Electronic Systems Program, National University of Singapore, E6 #05-4, 5 Engineering Drive 1, Singapore 117608, Singapore; 5 NUS Suzhou Research Institute (NUSRI), Suzhou Industrial Park, Suzhou 215123, China; 6 Faculty of Microsystem Electronics and Photonics, Wroclaw University of Science and Technology, 11/17 Janiszewskiego Str., Wroclaw 50-372, Poland; jan.dziuban@pwr.edu.pl

**Keywords:** triboelectric nanogenerator, self-powered, energy harvesting, internet of things (IoT)

## Abstract

Triboelectric nanogenerator (TENG) is a promising technology because it can harvest energy from the environment to enable self-sustainable mobile and wearable electronic devices. In this work, we present a flexible touch pad capable of detecting the contact location of an object and generating substantial energy simultaneously based on the coupling of triboelectric effects and electrostatic induction. The touch pad consists of Polytetrafluoroethylene (PTFE) thin film, multiple Aluminum (Al) electrodes and Polyethylene terephthalate (PET) layers, which can be achieved through low cost, simplified and scalable fabrication process. Different from the conventional multi-pixel-based positioning sensor (i.e., large array of sensing elements and electrodes), the analogue method proposed here is used to implement the positioning function with only four electrodes. Position location can achieve a detecting resolution of as small as 1.3 mm (the size of locating layer is 7.5 cm × 7.5 cm). For the energy harvesting part, a multilayer structure is designed to provide higher current output. The open circuit voltage of the device is around 420 V and the short circuit current can reach up to 6.26 µA with current density of 0.25 µA/cm^2^. The maximum output power obtained is approximately 10 mW, which is 0.4 mW/cm^2^. The flexibility and significantly reduced number of electrodes enable the proposed touch pad to be readily integrated into portable electronic devices, such as intelligent robots, laptops, healthcare devices, and environmental surveys, etc.

## 1. Introduction

In recent years, due to the increment in the number of portable electronics, energy harvesting from widely available mechanical energy sources for self-powered systems has attracted increasing attention [1,2,3]. Triboelectricity is a phenomenon where electrostatic charges are generated by physical contact of two different material surfaces. When the two materials are separated by mechanical force, the built-up electric potential can drive electrons to flow from the positive electrode to the negative electrode. Traditional materials cannot be uniformly contacted in the triboelectric process. Efforts to enhance the output performance of triboelectricity are not only about the selection of materials, but also the morphologies of material surfaces. Generally, surfaces with micro or nano roughness provide more contact area during the operation, resulting in more triboelectric charges. Physical and chemical techniques are adopted to modify material surfaces to enhance the roughness [4,5]. Based on the coupling of triboelectrification and electrostatic induction, triboelectric nanogenerators (TENGs) have recently been used to harvest mechanical energy from regular/irregular sliding, triggering, vibrations, and rotations. The materials normally used in the reported TENGs are mainly PTFE, PET, and Al foil, etc., which ensures the cost effectiveness of the system. In addition, the complex electric circuits can be avoided in these devices [6,7,8,9,10]. Therefore, in the electronics industry and intelligent robot applications, triboelectric-based self-powered sensors stand out from others because of their simple structure, widely available materials, high sensitivity, flexibility, light weight, portability, cost effectiveness and self-powered functionality [11,12,13,14,15].

Traditional pressure and tactile sensors can be classified into the following categories in terms of sensing mechanisms: piezoelectricity, capacitance, optics, and resistance [16,17,18,19]. However, the requirement of external power supply is one common limitation for most of these tactile sensors. In some extreme and remote locations, recharging or replacing batteries of sensors is expensive, inconvenient, or even impossible [20]. On the other hand, TENGs not only can be used for energy harvesting, but also can be used for self-generated sensing under mechanical triggering, which enables the integration of TENG-based sensors with portable electronic devices, wireless systems, and biomedical microsystems [21,22,23,24,25,26,27,28,29,30]. Based on the prediction from CISCO [31], there will be trillions of sensors distributed around the world by the year of 2020, which will consume a huge amount of energy. Thus, the next generation of sensors should be self-powered, free of maintenance, accurate and widely perceived. 

Due to the diversified working modes and excellent adaptability, TENGs can be fabricated into different forms to effectively harvest most of the traditional mechanical energy in daily life. Accordingly, triboelectric-based sensors can also be designed with different forms to achieve fast response, high sensitivity, and low detection limit [32,33,34,35,36,37]. For example, Shi et al. presented a flexible triboelectric-based microfluidic sensor to detect the applied pressure and monitor the finger bending motion [38]. Meng et al. developed a tactile sensor based on micro-patterned PDMS to achieve high output performance [39]. During the normal operation cycle, triboelectric charges on the dielectric surfaces due to contact electrification cause electrons to flow back and forth between the electrodes [40,41]. However, it is difficult to decouple the interference signals, and the simultaneous detection of complex information has not been realized. In the previous research, pixel-based digital methods have been widely used for high-resolution positioning. For example, to achieve a 5 × 5 positioning capability, 25 sensors and 50 electrodes need to be implemented. However, to achieve higher resolution, the number of sensors and electrodes in the device needs to be increased dramatically. This will increase the cost and introduce additional difficulties for data acquisition/processing, signal interference and fault analysis. The analogue method is a brand-new strategy in reducing the number of sensors and electrodes of the device. For example, Alam reported a breakdown spot position estimation technique based on the voltage ratio measurements [42]. Kim expanded a position-sensing mechanism in a 2D panel, drew a figure using the 2D ionic touch panel [43]. In the field of triboelectricity, Zhang’s group reported an analogue locating method for self-powered analogue smart skin, and a resolution of 1.9 mm was achieved with four electrodes [44]. Wang’s group reported a device with 4 × 4 array to sense location and pressure of object, demonstrating a resolution of 2 mm [45]. According to the reported results, self-powered systems based on TENG have been widely adopted for the characteristics of the pressure, attitude, and movement [46,47,48]. Increasingly theoretical analysis and process have been developed and the performance of the self-powered systems is also getting better and better [49,50,51,52,53,54]. Specifically, triboelectric sensors attract increasing research efforts due to its self-powered mechanism and low-cost solution for IoT applications [55,56,57].

**Figure 1:**
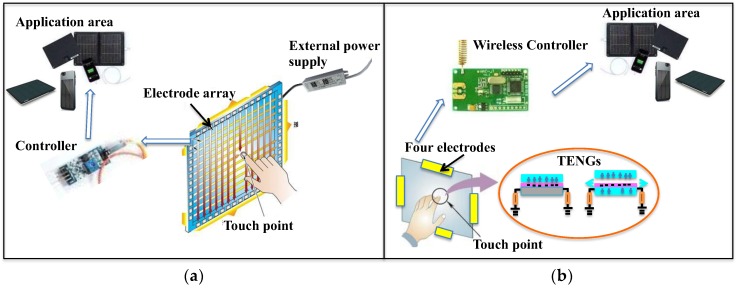
(**a**) The traditional tactile sensing system which requires external power supply and large number of sensors and electrodes to achieve high resolution; (**b**) Self-powered electronic system by the integration of flexible tactile sensor with only four electrodes and energy harvesting.

The traditional tactile sensing system needs external power supply, and to achieve high resolution, a large number of sensors and electrodes need to be implemented as shown in Figure 1a. Herein, we propose a flexible touch pad to realize both location detection with only four electrodes and energy-harvesting function for potential wireless transmission as shown in Figure 1b. It can detect the motion of an object through only four electrodes using the surface electrical properties. The output voltage can be obtained from the four electrodes, and then the ratio of the voltage can be calculated to determine the position where the object contacts. 

## 2. Materials and Device Configuration

**Figure 2:**
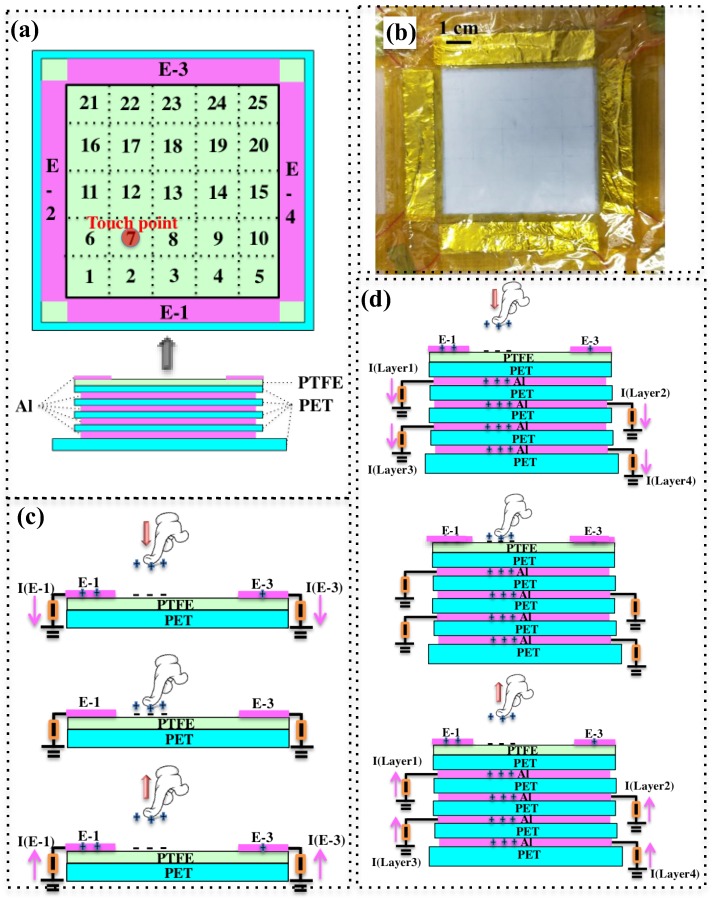
(**a**) Schematic illustration of the device from top view and side view, showing the four edge electrodes for position sensing and four stacking electrodes for energy harvesting. No. 1 to No. 25 denotes the virtual pixel (touch point) to facilitate finger contact test; (**b**) The photograph of the fabricated device; (**c**) The operation mechanism for position sensing of the device. Only the sensing part of the device is shown in the schematics; (**d**) The operation mechanism for energy harvesting of the device.

The device structure and photograph of the proposed touch pad are shown in Figure 2a,b. The device is fabricated in a square shape with symmetrical structure, which is composed of a locating/positioning part and an energy harvesting part. The locating part consists of a PET substrate, a PTFE layer and four Al electrodes as shown in Figure 2b. The size of the locating layer is 7.5 cm × 7.5 cm with four electrodes distributed on four edges. The energy harvesting part is formed by four 5 cm × 5 cm Al foil electrodes with three 7.5 cm × 7.5 cm thin PET layers in between and one 8.5 cm × 8.5 cm PET substrate at the bottom. To facilitate the operation and analysis of the touch pad through finger touching, the surface of the device is divided in to 25 virtual squares (5 × 5 pixels) with the size of 1 cm^2^, labeled from No. 1 to No. 25. The size of the virtual pixels is roughly the size of the effective area of finger contact. The four energy harvesting layers are labeled as Layer 1 to Layer 4 from the top to the bottom.

PTFE is one of the most negatively charged materials in triboelectric series with the electron affinity of −190 nC/J [58]. Finger wearing nitrite rubber glove is adopted as the positive material due to its good electropositive property. As shown in Figure 2c, after contacting with the PTFE layer, finger becomes positively charged and PTFE surface becomes negatively charged due to their difference in electron affinity. When the finger moves away from the PTFE surface, the negative charges on the PTFE surface repels electrons in the four electrodes to flow to ground through external circuits. When the finger contacts the PTFE surface again, the negative charges on the PTFE surface are balanced by the positive charges on the finger, thus electrons flow in a reverse direction. During the finger touch process, different amounts of charges are induced at the four electrodes depending on the position of the touch point. For example, when the touch point is closer to electrode 1 (E-1), the amount of induced charges on E-1 is larger than that on electrode 3 (E-3). The working principle of the energy harvesting part of the device is similar to the locating part, with charge induction on the bottom energy harvesting electrodes, as illustrated in Figure 2d. The multilayer bottom electrodes are fabricated to improve the overall energy harvesting performance of the device [59].

## 3. Characterization of Position Sensing

**Figure 3:**
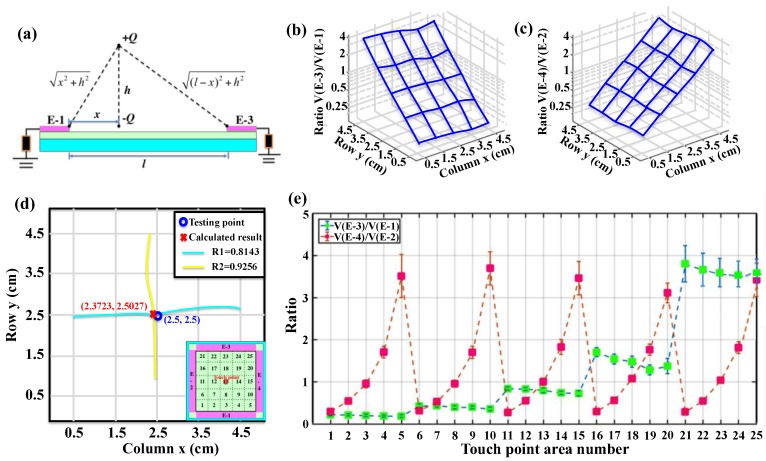
(**a**) The electrostatic analysis of the contact process. The average ratios of (**b**) *V*(E-3)/*V*(E-1) and (**c**) *V*(E-4)/*V*(E-2) at each touch point in semi-log z-axis plot; (**d**) Testing result of touch point (2.5, 2.5), indicating the calculated position and the actual position are highly matched, with deviation less than 1.3 mm. The inset shows the location of the touch point on the device; (**e**) The average values and the standard deviation values of the ratios (*V*(E-3)/*V*(E-1) and *V*(E-4)/*V*(E-2)) in a sequential order.

To obtain the location of the touch point by analogue method, the relationship of output voltage from the four electrodes is first studied. The theoretical analysis of electrostatic induction during the contact process is illustrated in Figure 3a. During the process of the finger approaching and leaving the device surface, the electrical field on the four electrodes changes accordingly.

If the electric potential of ground and infinite distance is assumed to be of 0 V, then the electric potential of a point charge can be written as

(1) $$U=k\frac{Q}{r}$$

where *Q* is the amount of charge, *r* is the distance to the point charge, and *k* is the Coulomb’s constant (*k* = 8.9875 × 10^9^ N m^2^ C^−2^).

The distance between two opposite electrodes (E-1 and E-3) is assumed to be *l*. After contacting with the PTFE surface, the finger with charge of −*Q* moves away from the PTFE surface with a distance of *h*. The touch point on PTFE surface is with charge of −*Q* correspondingly. If the distance between the touch point and E-1 is *x*, then the distance between the touch point and E-3 is *l* − *x*. Thus, the electric potentials of the E-1 and E-3 (*V*(E-1) and *V*(E-3)) can be expressed as

(2) $$\left\{ \begin{array}{l} V(E-1)=k\frac{Q}{\sqrt{x^{2}+h^{2}}}-k\frac{Q}{x}\\ V(E-3)=k\frac{Q}{\sqrt{{\left(l-x\right)}^{2}+h^{2}}}-k\frac{Q}{l-x} \end{array}\right.$$

Their ratio can be derived as

(3) $$\frac{V(E-3)}{V(E-1)}=\frac{k\frac{Q}{\sqrt{{\left(l-x\right)}^{2}+h^{2}}}-k\frac{Q}{l-x}}{k\frac{Q}{\sqrt{x^{2}+h^{2}}}-k\frac{Q}{x}}=\frac{\frac{1}{\sqrt{{\left(l-x\right)}^{2}+h^{2}}}-\frac{1}{l-x}}{\frac{1}{\sqrt{x^{2}+h^{2}}}-\frac{1}{x}}$$

The ratio is dependent on the position of the touch point. When *h* is zero, *V*(E-1) = *V*(E-3) = 0 because the electrical filed is located between the finger and PTFE film. With the increasing of *h*, the electrode voltage approaches *V*(E-1) = −*kQ*/*x* and *V*(E-3) = −*kQ*/(*l* − *x*). These trends suggest that the final relative position of finger and PTFE film will affect the output voltage. Therefore, when *h* is much larger than *l*, the ratio can be simplified to

(4) $$\frac{V(E-3)}{V(E-1)}\approx \frac{\frac{1}{1-x}}{\frac{1}{x}}=\frac{x}{1-x}$$

Due to the symmetrical structure design, the same relationship between *V*(E-2) and *V*(E-4) can also be obtained (*y* is the distance from the touch point to E-2).

(5) $$\frac{V(E-4)}{V(E-2)}\approx \frac{\frac{1}{1-y}}{\frac{1}{y}}=\frac{y}{1-y}$$

To obtain a two-dimensional location for the contact, two voltage ratios of opposite electrodes (denoted as *V*(E-3)/*V*(E-1) and *V*(E-4)/*V*(E-2)) are measured with a 5 × 5 test point (Figure 3b,c). Measurement for each virtual pixel is repeated for 40 times to calculate the average value as the standard contact point in the square area. For each point, 40 groups of the peak output voltages are obtained in advance. Then the ratios of *R*1 = *V*(E-3)/*V*(E-1) and *R*2 = *V*(E-4)/*V*(E-2) of each group are calculated using MATLAB. The average values of the calculated R1 and R2 for each touch point are listed in Table 1 and Table 2, respectively. *R*1 and *R*2 monotonously increase and the voltage ratio changes show good resolution.

The ratios of *R*1 remain relatively the same when the touch points are at the same row since the distances to E-1 are the same. The distances to E-3 can be seen from Figure 3b, which matches with the relationship in the hypothesis. Similarly, the ratios of *R*2 remain relatively the same when the touch points are at the same column. The distances to E-4 can be seen from Figure 3c. All the average values and the standard deviation values of the ratios are plotted in Figure 3e. The ratios obtained can be divided into five similar values for both *R*1 and *R*2 when the touch point changes, i.e., 0.2, 0.4, 0.8, 1.5 and 3.5 for *R*1, and 0.28, 0.53, 1, 1.75 and 3.5 for *R*2, respectively. The five values are slightly different for the two voltage ratios due to the fabrication and assembling deviations. For *R*1, considering both the average value and the standard deviation, the ratios at different rows can be separated, which is the same for *R*2 at different columns.

To characterize the performance of the locating capability of the device, the touch point No. 13 at (2.5, 2.5) is tested firstly, as shown in Figure 3d. The ratios obtained from the measured output voltages are *R*1 = 0.8143 and *R*2 = 0.9256. By using the ratio map in Figure 3b,c for interpolation, two curves corresponding to *R*1 = 0.8143 and *R*2 = 0.9256 are plotted and the intersection point can be calculated by MATLAB as (2.3723, 2.5027). The distance deviation between the actual touch point and the calculated point is 1.277 mm. Similarly, through experimental testing and calculation by MATLAB, the distance deviations of all twenty-five points are between 0.021–1.293 mm. Therefore, the position-sensing resolution of the device is ~1.3 mm.

**Figure 4:**
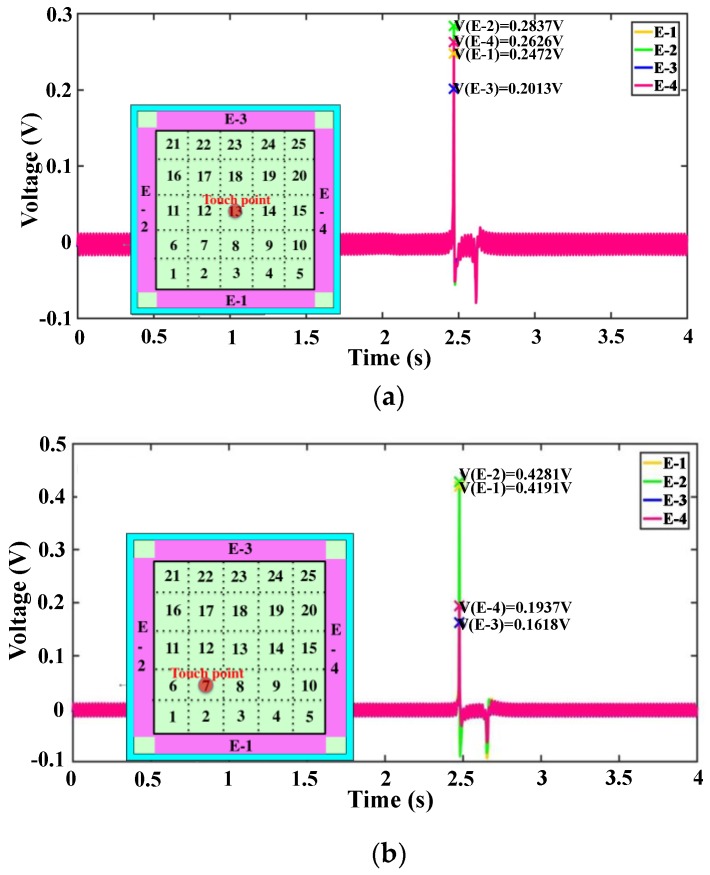
Output voltages of the four electrodes when testing is performed at (**a**) point No. 13 and (**b**) point No. 7.

The four output voltages remain 0 V when no contact with the device happens. Once a contact with the device surface happens, output voltages are formed simultaneously on the four electrodes. The peaks of the four output voltages can be detected easily. Examples of detecting the output voltages from four electrodes with touch point No. 13 and touch point No. 7 are shown in Figure 4.

**Figure 5:**
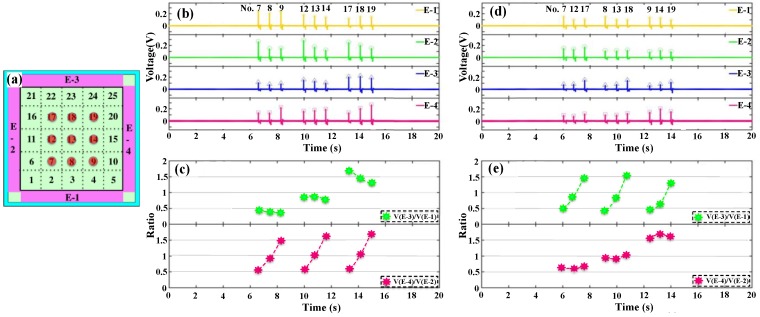
(**a**) Schematic illustration of the nine touch points at the center of the device; (**b**) The output voltage values and (**c**) the ratios (*V*(E-3)/*V*(E-1) and *V*(E-4)/*V*(E-2)) of the nine continuous touches in the direction from E-2 to E-4; (**d**) The output voltage values and (**e**) the ratios (*V*(E-3)/*V*(E-1) and *V*(E-4)/*V*(E-2)) of the nine continuous touches in the direction from E-1 to E-3.

**Figure 6:**
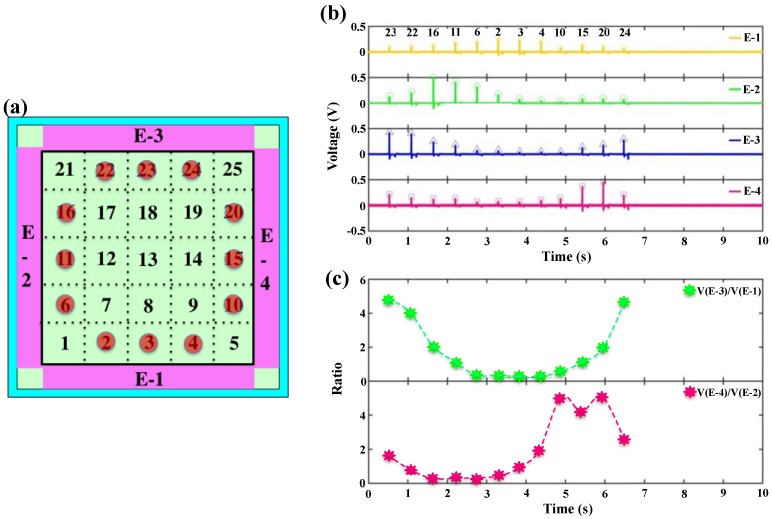
(**a**) Schematic illustration of the twelve tested points forming a circle in the direction from No. 23 to No. 24. (**b**) The output voltages and (**c**) the ratios of the twelve continuous touche.

Measurements on different touch points are also conducted sequentially, with the potential application for writing pad after further optimization. Figure 5 and Figure 6 shows the measurement results of the middle nine points and twelve circular points, respectively.

Figure 5a shows the location of the continuous nine tested points where the contacting frequency can be up to 2 Hz. Figure 5b,c shows the output voltages and the ratios of the nine continuous touch points at the center (No. 7 to No. 19) with the contacting sequence from E-2 to E-4. Figure 5d,e show the output voltages and the ratios of the nine continuous touch points at the center (No. 7 to No. 19) with the contacting sequence from E-1 to E-3. From the results, the ratios obtained maintain the regular pattern and match with the theoretical analysis for both testing sequence, indicating the excellent stability of the device. Corresponding to the points in Figure 5c,e, the calculated values are listed in the Table 3 and Table 4. respectively. Next, the circle-pointing experiment is performed, with the location of the touch points shown in Figure 6a. The twelve continuous touch points are tested from No. 23 (2.5, 4.5) to No. 24 (3.5, 4.5) in anticlockwise manner. Figure 6b,c shows the output voltages and the ratios of the twelve continuous touch points forming a circle. Corresponding to the points in Figure 6c, the calculated values of the ratio are listed in Table 5.

**Figure 7:**
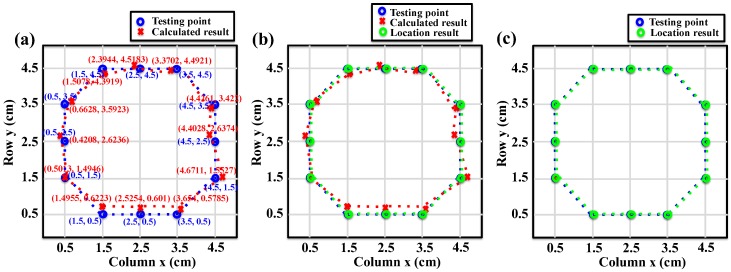
The actual position of the twelve testing points (blue), the calculated results (red) and the final location results (green) of the circle-pointing experiment. (**a**) Comparison of the actual position of the testing points with the calculated results; (**b**) Comparison of the actual position of the testing points with the calculated results and the final location results; (**c**) Comparison of the actual position of the testing points and the final location results, indicating perfect reconstruction of the testing positions.

From the generated output voltages and their ratios, the position of the twelve touch points can be reconstructed. The actual testing points, the calculated results and the location results with circular touch points are compared in Figure 7. Figure 7a compares the calculated results with the actual testing points, which shows the circular shape of the testing points is preserved only with small deviations. Figure 7b shows the actual testing points, calculated results, and location results (the center of the touch point area). Figure 7c compares the location results with the actual testing points. When the calculated result of a touch point is within the area of a virtual pixel, the position of the touch point (location result) will be located to the middle of the corresponding virtual pixel. It can be seen that the location results match perfectly with the actual testing results, showing the capability of the touch pad for position location.

## 4. Characterization of Energy Harvesting

**Figure 8:**
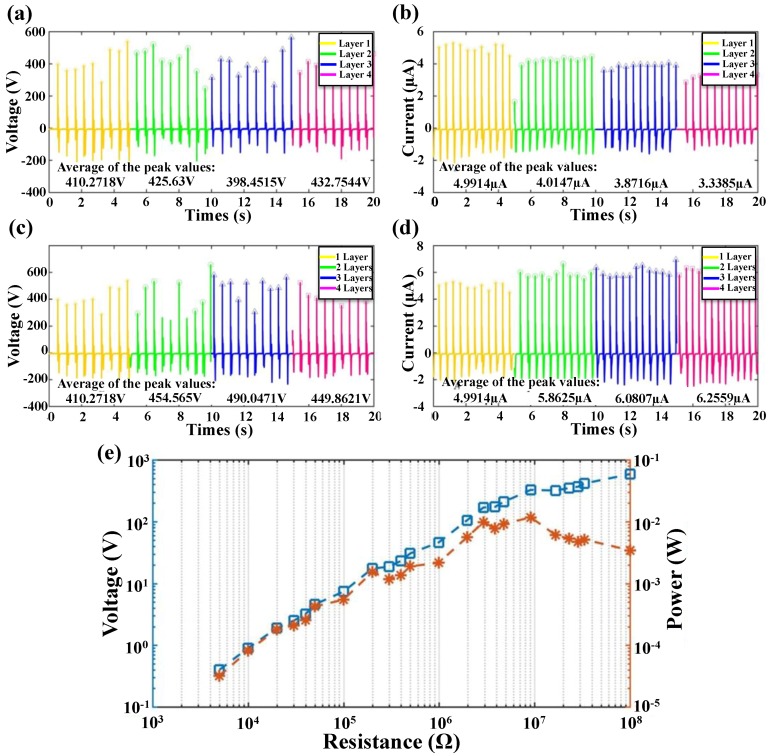
(**a**) The open circuit voltage and (**b**) short circuit current from the four energy harvesting layers (Layer 1 to Layer 4) separately. To compare the differences of the signals from four layers, a subsection test is applied from Layer 1 to Layer 4, with each test of 5 s; (**c**) The open circuit voltage and (**d**) short circuit current from the four energy harvesting layers with parallel connection of different layers from top to bottom; (**e**) The output voltage and power from the four parallel-connected layers on different external loads.

In the design of the touch pad, the bottom four thin PET/Al layers are fabricated for energy harvesting. The four energy harvesting layers (one thin PET film and one Al electrode) are labeled as Layer 1 to Layer 4 from the top to the bottom. The open circuit voltage and the short circuit current of the four energy harvesting layers are measured and compared with finger tapping. Both the voltage and current are measured in separate and parallel-connected manner. In terms of the output from one single layer, both the open circuit voltage and the short circuit current decrease slightly from top to bottom as shown in Figure 8a,b, due to the increment of distance between the layer and the charged surface. As shown in Figure 8c,d, when different layers are connected in parallel, the open circuit voltage remains relatively the same of ~420 V, while the short circuit current increases from 4.9914 µA to 6.2559 µA with more layers connecting in parallel. Thus, the four-layer structure can provide a better energy harvesting capability with current increment of 25%.

Therein, based on the current and voltage measured from the device, the current density and power density can be calculated by the following equations:

(6) $$J_{d}=I_{R}/S$$

(7) $$P_{d}=U_{R}I_{R}/S$$

where *J_d_* and *P_d_* are the current density and power density, respectively. *I_R_* and *U_R_* are the current and voltage provided by the four-layer structure, respectively. *S* is the effective working area which is 25 cm^2^. When the short circuit current is 6.2559 µA, the corresponding current density is about 0.25 µA/cm^2^.

The output voltage and power from the four parallel-connected layers on different external loads are shown in Figure 8e. When the voltage and current are about 300 V and 33.3 µA, respectively, the output power reaches its maximum value of 10 mW at 9 MΩ. Combined with area of 25 cm^2^, the power density can be calculated as 0.4 mW/cm^2^.Therefore, the enhanced energy harvesting capability of the device can provide energy supply potentially for the wireless communication between the device and the commercial portable circuits.

As mentioned above, we have demonstrated the methodology of a flexible touch pad for sensing the contact location of an object and supplying enough energy for potential wireless transmission. According to the generated output voltage and power from the energy harvesting part, the device has reached the power required for wireless transmission. For the next step of future work, energy storage module and the wireless transmission module will be further assembled into the device to realize the integration and practicability of the device.

## 5. Conclusions

In summary, a flexible touch pad is proposed and investigated with theoretical modeling and experimental characterization. With only four sensing electrodes on top, it can detect the contact location of an object with detecting resolution as small as 1.3 mm. With the multilayer structure design for energy harvesting, enhanced current output can be achieved. Under finger tapping, the touch pad can generate open circuit voltage of ~420 V and short circuit current of 6.26 µA with current density of 0.25 µA/cm^2^. The maximum output power obtained is approximately 10 mW, which is 0.4 mW/cm^2^. The touch pad with only four electrodes for position location shows great potential for future applications to increase detection resolution with low cost and low complexity, because the theoretical estimation of resolution is much higher. In many fields such as artificial intelligence and bionics, the self-powered devices without external power supply show bright prospects. We anticipate further optimization would enable the self-powered touch pad to be easily integrated into portable electronic devices.

## Figures and Tables

**Table 1:** Voltage ratios calculated (*R*1) using MATLAB.

	*x* = 0.5	*x* = 1.5	*x* = 2.5	*x* = 3.5	*x* = 4.5
*y* = 4.5	3.8004	3.6505	3.3684	2.889	3.1085
*y* = 3.5	1.703	1.5404	1.4802	1.2836	1.3732
*y* = 2.5	0.8411	0.8294	0.7971	0.7378	0.7274
*y* = 1.5	0.4265	0.4359	0.397	0.3972	0.3578
*y* = 0.5	0.2225	0.2152	0.2044	0.1887	0.1876

**Table 2:** Voltage ratios calculated (*R*2) using MATLAB.

	*x* = 0.5	*x* = 1.5	*x* = 2.5	*x* = 3.5	*x* = 4.5
*y* = 4.5	0.2867	0.5472	1.0378	1.8145	3.4459
*y* = 3.5	0.2934	0.5613	1.0762	1.7614	3.1086
*y* = 2.5	0.2763	0.5526	1.0027	1.8276	3.4593
*y* = 1.5	0.3163	0.5312	0.9572	1.6986	3.6931
*y* = 0.5	0.2976	0.5463	0.9545	1.7053	3.5104

**Table 3:** Voltage ratios of No. 7 to No. 19 from E-2 to E-4 calculated using MATLAB.

Voltage Ratios	No. 7	No. 8	No. 9	No. 12	No. 13	No. 14	No. 17	No. 18	No. 19
*V*(E-3)/*V*(E-1)	0.4512	0.3805	0.386	0.8588	0.8728	0.7605	1.7273	1.455	1.2955
*V*(E-4)/*V*(E-2)	0.5299	0.9294	1.4623	0.5837	1.0252	1.6991	0.5472	1.0273	1.7275

**Table 4:** Voltage ratios of No. 7 to No.19 from E-1 to E-3 calculated using MATLAB.

Voltage Ratios	No. 7	No. 12	No. 17	No. 8	No. 13	No. 18	No. 9	No. 14	No. 19
*V*(E-3)/*V*(E-1)	0.5218	0.8475	1.4187	0.4418	0.8143	1.5311	0.4536	0.6301	1.2824
*V*(E-4)/*V*(E-2)	0.6295	0.6035	0.6796	0.9333	0.9014	1.0386	1.5705	1.6925	1.6028

**Table 5:** Voltage ratios of No. 23 to No. 24 with a circle.

Voltage Ratios	No. 23	No. 22	No. 16	No. 11	No. 6	No. 2	No. 3	No. 4	No. 10	No. 15	No. 20	No. 24
*V*(E-3)/*V*(E-1)	4.7561	3.9515	1.9747	1.0719	0.4253	0.2991	0.2153	0.2454	0.583	1.098	1.9238	4.5863
*V*(E-4)/*V*(E-2)	1.6412	0.7526	0.2696	0.3359	0.2296	0.5009	0.9345	1.9636	4.9517	4.1615	4.9946	2.5985
